# Shared neural codes for visual and semantic information about familiar faces in a common representational space

**DOI:** 10.1073/pnas.2110474118

**Published:** 2021-11-03

**Authors:** Matteo Visconti di Oleggio Castello, James V. Haxby, M. Ida Gobbini

**Affiliations:** ^a^Center for Cognitive Neuroscience, Dartmouth College, Hanover, NH 03755;; ^b^Department of Experimental, Diagnostic and Specialty Medicine, University of Bologna, 40138 Bologna, Italy;; ^c^Cognitive Science Program, Dartmouth College, Hanover, NH 03755

**Keywords:** familiar face processing, face recognition, social perception, person knowledge, brain decoding

## Abstract

Processes evoked by seeing a personally familiar face encompass recognition of visual appearance and activation of social and person knowledge. Whereas visual appearance is the same for all viewers, social and person knowledge may be more idiosyncratic. Using between-subject multivariate decoding of hyperaligned functional magnetic resonance imaging data, we investigated whether representations of personally familiar faces in different parts of the distributed neural system for face perception are shared across individuals who know the same people. We found that the identities of both personally familiar and merely visually familiar faces were decoded accurately across brains in the core system for visual processing, but only the identities of personally familiar faces could be decoded across brains in the extended system for processing nonvisual information associated with faces. Our results show that personal interactions with the same individuals lead to shared neural representations of both the seen and unseen features that distinguish their identities.

## Significance

Our brain processes faces of close others differently than faces of visually familiar individuals. While both types of faces activate similar visual areas, faces of close others activate areas involved in processing social and semantic information. Here, we used between-subject linear classifiers trained on hyperaligned brain data to investigate the neural code for visual and semantic information about familiar others. The identity of both visually and personally familiar faces could be decoded across participants from brain activity in visual areas. Instead, only the identity of personally familiar faces could be decoded in areas involved in social cognition. Our results suggest that individually distinctive information associated with familiar faces is embedded in a neural code that is shared across brains.

Face recognition is essential for effective social interactions. When we see a familiar face, we spontaneously retrieve person knowledge and the position occupied by that familiar individual in our social network. This information sets us up for the most appropriate behavior with that specific individual. The importance of familiar faces for social interactions is reflected in the way the human brain processes these stimuli. Familiar faces are processed in a prioritized way with faster detection even in suboptimal conditions (1, 2). Familiarity associated with faces warps their visual representation (3) and can result in a more homogenous representation across the visual field in areas with retinotopic organization (4). Recognition of familiar faces entails processing not only their visual appearance but also retrieval of person knowledge and an emotional response (5–10). Different parts of the distributed neural system for face perception contribute to these processes (5, 10, 11). The core system for face perception processes visual appearance, resulting in view-invariant representations of identity in anterior temporal and inferior frontal face areas (12–14). The extended system for face perception plays a role in extracting semantic information from faces as well as emotional responses (5, 11, 15, 16).

Here, we investigated the neural codes for high-level visual and semantic information about personally familiar faces. Specifically, we asked whether these codes are supported by a common set of basis functions that are shared across people who are personally familiar with the same individuals. We measured patterns of brain activity with functional magnetic resonance imaging (fMRI) while participants viewed images of personally familiar faces and faces of strangers who were only visually familiar. We used hyperalignment to derive a set of basis functions that align brain response patterns in a common, high-dimensional information space (17–19). Hyperalignment transformation parameters were based on participants’ brain activity measured while watching *The Grand Budapest Hotel* (20), an engaging comedy-drama with rich characterizations of several individuals. We found that these basis functions capture shared representations of visual appearance in the core system for both personally familiar faces and visually familiar faces of strangers. Surprisingly, we also found basis functions that capture shared representations of personally familiar others, but not visually familiar strangers, in extended system areas that are associated with representation of person knowledge, theory of mind, and emotion. Importantly, these basis functions are derived from brain responses to the movie and are, thus, not specific to the familiar individuals whose faces were the experimental stimuli. These results show that the face processing system encodes both visual and nonvisual high-level semantic information about personally familiar others in a neural information space that is not specific to a given set of faces and that is shared across brains.

## Results

**Fig. 1.**
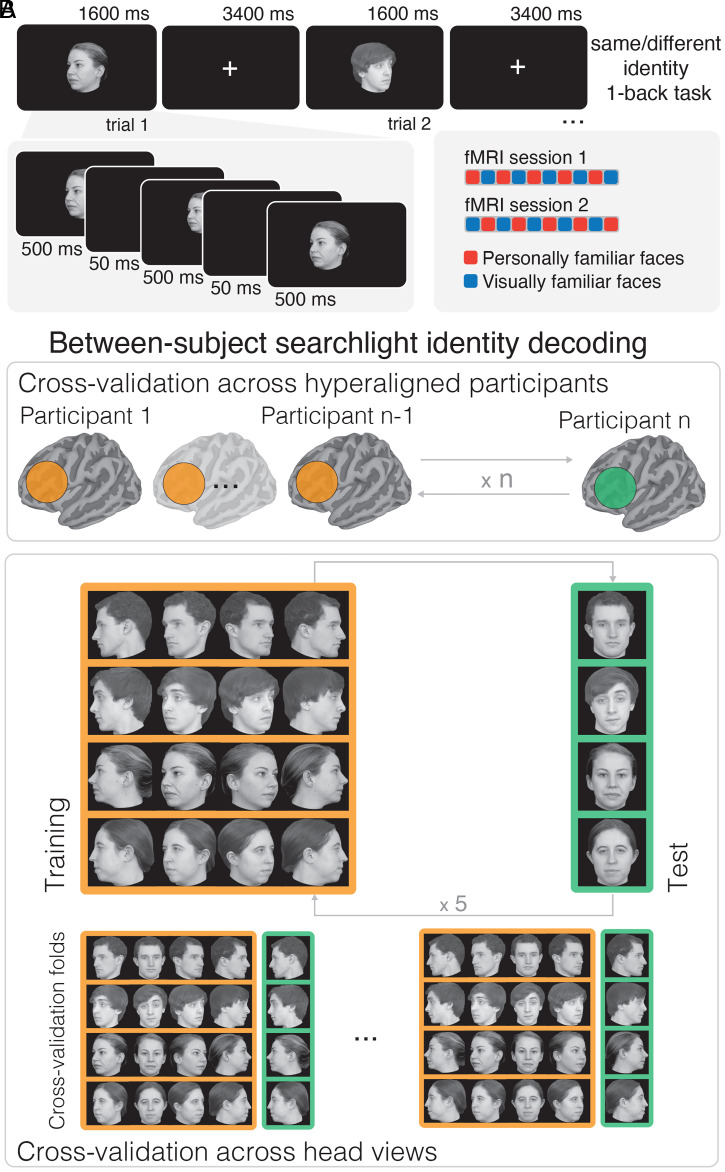
Experimental paradigm and between-subject identity decoding analyses. (*A*) Experimental paradigm with two example trials. In each trial, an image was presented for 1,600 ms (*Upper*). To prevent visual adaptation, the same image was repeated three times for 500 ms with random jitters in size and location between repetitions (*Lower*). After each trial, participants reported whether the identity presented in the current trial was the same or different as the previous trial (1-back repetition detection on identity) with a button press at every trial. The familiarity of the stimuli was blocked within runs and counterbalanced across sessions (*Lower Right*; see *Materials and Methods* for more details). (*B*) We performed between-subject identity decoding on hyperaligned data by cross-validating across participants and head views. The hyperaligned functional data were divided into a training set with n − 1 participants and a test set with the left-out participant (*Top*). Then, within each surface searchlight, a linear classifier was trained on samples from the n − 1 participants to distinguish the four identities in four head-views (*Bottom*, orange shaded box). The classifier was tested on the left-out head-view from the left-out participant (green shaded box). The process was repeated exhaustively for all five head-views (see example splits at the bottom) and for all participants. Thus, identity classifiers were tested for generalization across both participants and head views.

We measured patterns of brain responses to images of the faces of four personally familiar individuals and four individuals who were only visually familiar (each presented in five different head views; Fig. 1*A*) in a group of 14 participants who had known each other for over 2 y. Participants were graduate students, and the personally familiar faces were four other students in the same PhD program. Personally familiar individuals were rated as highly familiar by the participants (see *SI Appendix*, Fig. S1 and *Supplementary Information* for quantitative metrics of familiarity). The four individuals who were only visually familiar were previously unknown. Participants became visually familiar with these faces during an extensive behavioral training session a day prior the fMRI experiment (average face recognition accuracy during the training session was 97.9% [95% bootstrapped CI: 96.9, 98.6]; see *Materials and Methods* for more details and *SI Appendix*, Fig. S1).

**Fig. 2.**
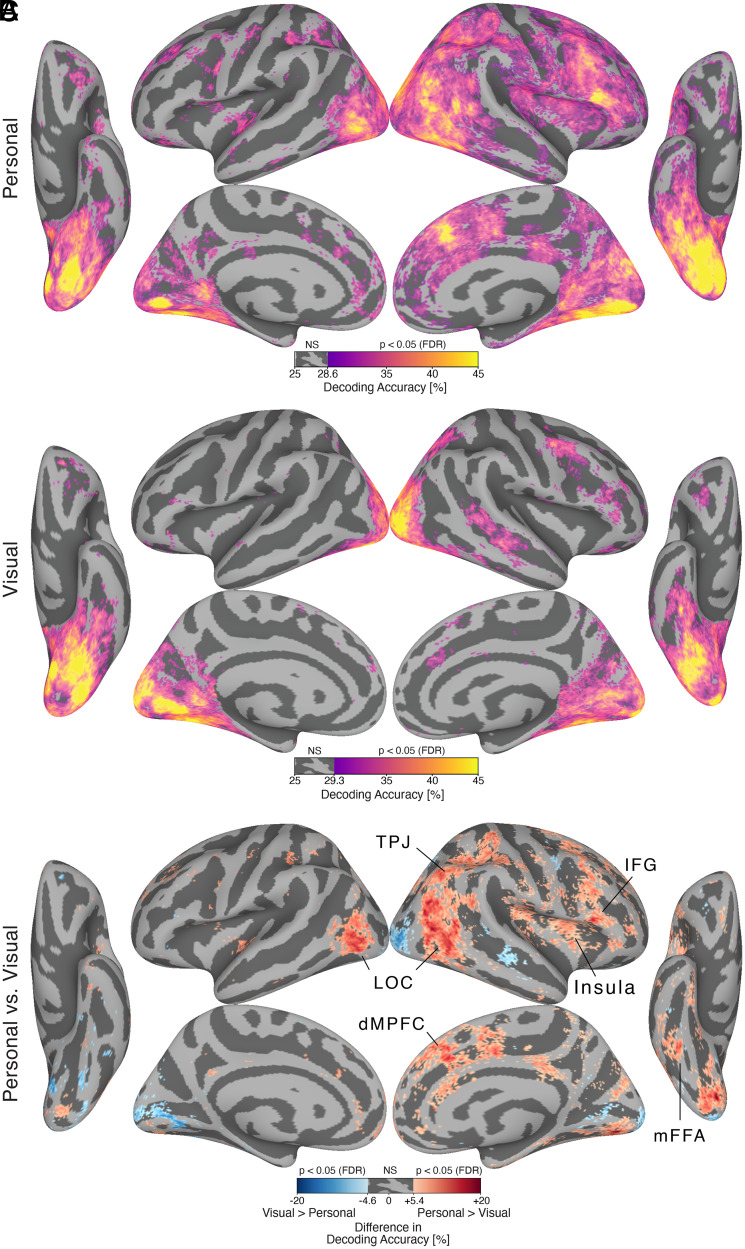
Decoding identity across participants with surface-searchlight, between-subject MVPC after hyperalignment. (*A*) Brain map showing decoding accuracy for personally familiar faces. (*B*) Brain map showing decoding accuracy for visually familiar faces. Both maps are thresholded at *P* < 0.05, one-sided, after permutation testing and FDR correction (Benjamini-Hochberg). “NS” on the colorbar indicates the range of nonsignificant accuracy values greater than 25%. (*C*) Difference in decoding accuracy between personally familiar faces and visually familiar faces. Red vertices indicate higher decoding accuracy for personally familiar faces (Personal > Visual). Blue vertices indicate higher decoding accuracy for visually familiar faces (Visual > Personal). The map is thresholded at *P* < 0.05, two-sided, after permutation testing and FDR correction (Benjamini-Hochberg). For personally familiar faces, significant between-subject decoding accuracy was present across the core system: bilateral OFA and fusiform gyrus, right ATL (ventral core system); right pSTS, mSTS, and anterior STS (dorsal core system); and right IFG (anterior core system). Significant decoding accuracy was also present in areas of the extended system: right TPJ and MPFC(theory of mind areas), bilateral precuneus, and right insula. For visually familiar faces, significant between-subject decoding accuracy was limited to areas of the core system: bilateral OFA and fusiform gyrus, right mSTS, and right IFG. Between-subject decoding accuracy for personally familiar faces was higher than for visually familiar faces in large portions of the face processing network. Higher decoding accuracy for personally familiar faces was present in areas of the core system such as bilateral lateral occipital cortex (LOC), right mFFA, and right IFG, and areas of the extended system such as right TPJ, precuneus, MPFC, and insula.

We used fMRI data collected while participants watched a movie (*The Grand Budapest Hotel*) to derive a common model of information spaces with hyperalignment (17). For each participant, hyperalignment calculates transformations that remix that individual’s cortical vertices into the model space dimensions. These dimensions capture response basis functions that are shared across brains, affording markedly stronger between-subject decoding of brain response patterns (Fig. 2 and *SI Appendix*, Fig. S2). Because these transformations are performed on cortical vertices, they can be applied to new responses in the same participant to model responses for new stimuli that were not used in their derivation. These transformations derived from independent movie-viewing data were used to project brain responses to the face images in the familiar face perception experiment into the shared model information space.

We performed separate between-subject multivariate pattern classifications (MVPC) of responses to personally familiar faces and the faces of visually familiar strangers. To analyze head-view-invariant representation of identity, support vector machine (SVM) classifiers of identity were trained on responses to images of four head views and tested on the left-out head view (Fig. 1*B*).

**Fig. 3.**
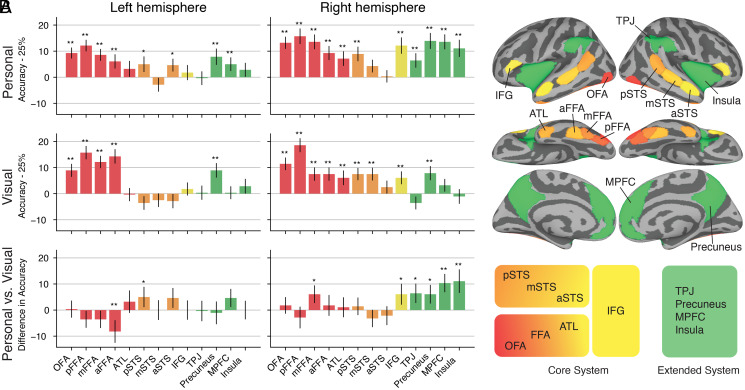
Decoding identity across participants in areas of the core and extended system with between-subject MVPC after hyperalignment. (*A*) The barplots show between-subject classification accuracy within ROIs in the core system (red, orange, and yellow bars) and in the extended system (green bars). Accuracy values are centered around chance level (25%). Error bars show SEM (SE, *n* = 70 cross-validation folds); * indicates significance at *P* < 0.05 without FDR correction; ** indicates significance at *P* < 0.05 with FDR correction (Benjamini-Hochberg). *P* values were estimated with permutation testing (one-sided for *Top* and *Middle*, two-sided for *Bottom*; see *Materials and Methods* for details). *Top*: personally familiar faces. *Middle*: visually familiar faces. *Bottom*: difference in accuracy between personally familiar faces and visually familiar faces. (*B*) Brain map showing the ROIs whose accuracy values are plotted in *A*. ROIs for the core system were determined with a dynamic face localizer. ROIs of the extended system were determined with an anatomical parcellation (see *Materials and Methods*). The diagram at the bottom shows the distinction in ventral, dorsal, and anterior core system and extended system, as proposed in ref. 13. Decoding of identity for both personally and visually familiar faces was significant in most areas of the core system, including FFA, ATL, and IFG (red and yellow bars). However, in extended system areas (green bars), decoding of identity was present only for personally familiar faces (except in bilateral precuneus).

Between-subject classification of identity revealed an extensive set of cortical areas with significant decoding accuracies (Figs. 2 and 3). Whole-brain searchlight analyses (Fig. 2 and *SI Appendix*, Fig. S3) revealed significantly higher decoding accuracies for personally familiar faces than for visually familiar faces in the bilateral lateral occipital cortex, right middle fusiform face area (mFFA), and right inferior frontal gyrus (IFG), as well as in right temporoparietal junction (TPJ), right insula, and right dorsal and ventral medial prefrontal cortex (MPFC). Region-of-interest (ROI) analyses (Fig. 3 and *SI Appendix*, Figs. S4–S6) corroborated these findings and showed significant decoding for both visually and personally familiar identities in core areas in bilateral occipital face area (OFA), posterior FFA (pFFA), mFFA, and anterior FFA (aFFA) and in right posterior superior temporal sulcus (pSTS), right anterior temporal lobe (ATL), and right IFG, as well as in extended areas in bilateral precuneus. Decoding accuracies for personally familiar faces were significantly higher than accuracies for visually familiar faces in two areas of the right extended system—the MPFC and the insula—and, at a lower threshold, in the right TPJ and precuneus (two additional extended system area), the right mFFA, the right IFG, and the left pSTS. Significantly higher decoding accuracy for visually familiar faces than personally familiar faces was found only in the left aFFA. A right hemispheric bias was evident in the ROI analyses for both personally familiar and visually familiar faces (*SI Appendix*, Fig. S7).

Identity decoding accuracies for personally familiar faces were significant across the core and extended systems. In the core system, decoding was significant in the ventral core system (bilateral OFA; bilateral pFFA, mFFA, and aFFA; and the right ATL), in the dorsal core system (bilateral pSTS), and in the anterior core system (right IFG). Importantly, we found significant identity decoding of personally familiar face identity in nonvisual areas of the extended system (right TPJ, bilateral MPFC, bilateral precuneus, and right insula).

By contrast, significant identity decoding accuracies for visually familiar faces of strangers were limited to areas of the core system and the precuneus (see Figs. 2*B* and 3*A*). Decoding accuracies were significant in the ventral core system (bilateral OFA; bilateral pFFA, mFFA, and aFFA; and the right ATL), in the dorsal core system (right pSTS and middle STS [mSTS]), and in the anterior core system (right IFG).

## Discussion

This study provides direct evidence of shared representations of the distinctive visual appearance of familiar faces and individual-specific person knowledge associated with personally familiar others. We show that individually distinctive representations of familiar faces are embedded in a neural code that is shared across brains. Two types of familiarity were investigated: visual familiarity developed in the laboratory and with no associated semantic information and personal familiarity that results from direct and protracted social interactions over time (on average 3.2 y ± 1.4 SD). Personally familiar faces, unlike faces that are only visually familiar, have robustly distinct representations in areas of the extended system, notably in areas associated with person knowledge, theory of mind, social cognition, and emotional responses. These nonvisual areas include the TPJ, MPFC, precuneus, and insula (11, 15, 16, 21–23). This work both replicates and extends previous findings on the activation of the extended system by analyzing the information that is encoded in brain activity of the extended system areas. Here, we show that these activations encode high-dimensional representations for personally familiar others and that the neural code for these representations is shared across the brains of people who are personally familiar with the same friends and acquaintances.

By creating a common representational space using hyperalignment, we were able to decode identity information across participants. Between-subject classifiers are a stringent test to determine whether the information about the stimuli and the way this information is encoded in brain activity are shared across participants (17–19, 24). These classifiers are trained on data from n − 1 subjects and tested on the left-out nth subject. In order for the classifier accuracy to be significantly above chance, information about the stimuli must be encoded in a similar format in the training subjects’ brains and in the test subject’s brain. Thus, our between-subject decoding results show that both the visual and semantic information encoded in idiosyncratic fine-scale patterns of brain responses are shared across people who are personally familiar with the same identities.

Decoding accuracy of identity was high for both personally familiar and visually familiar faces in the early core system for visual processing of faces, with higher decoding of personally familiar faces in patches near the functionally defined OFA and the right fusiform gyrus (Fig. 2*C*). These findings indicate that learning associated with the development of personal familiarity modifies the representation of faces at multiple levels. More robust representation in areas for visual and semantic representation of others may underlie, in part, the behavioral advantages for detection and recognition of personally familiar faces (1, 2, 4, 25–27). Conversely, alterations in structural and functional connectivity between these visual and semantic areas may underlie changes in face processing abilities with aging and deficits such as prosopagnosia (28, 29).

Decoding accuracy of identity was high for personally familiar faces in areas associated with person knowledge and processing of social information (5, 9, 16, 30, 31). This finding suggests that participants developed shared person knowledge about the personally familiar identities even if they had unique real-life interactions with those identities and that a shared code for this information is embedded in areas of the extended system for face perception. Shared person knowledge was reflected in similar patterns of brain activity in a common high-dimensional representational space across participants. This high-dimensional information space might encode a person knowledge information space. Our knowledge about an identity includes both episodic long-term memories and semantic attributes associated with a specific individual, such as personal traits, attitudes, opinions, and position in the social network (16, 32–34). Constellations of these attributes for individual persons can be thought of as vectors in a high-dimensional information space that captures the complexity of person knowledge. In this space, each individual has a unique person knowledge vector that distinguishes their identity. Some features can be common across identities (for example, similar episodic memories associated with different identities), but each identity is defined in aggregate by a unique constellation of multiple features. In order to be represented in brain activity, such a high-dimensional person knowledge information space should map onto a high-dimensional neural information space. Our work suggests that this high-dimensional neural information space exists. Future work will need to investigate the structure and content of the shared person knowledge and how it maps onto the high-dimensional neural information space. In this study, shared person knowledge could be the result of the shared experiences that the group of participants (PhD students in the same program) had on a daily basis in a group setting. Alternatively, the person knowledge shared by the participants might contain the more stable attributes and traits associated with the personally familiar identities.

Our study provides evidence for the critical importance of using personally familiar faces to investigate the full potential and scope of the face processing system. Previous work has shown that personal familiarity is not equivalent to familiarity developed over passive exposure, such as familiarity with famous individuals (6–8). Studies comparing brain responses to personally familiar faces versus famous faces found stronger activation for personally familiar faces in areas of the extended system such as TPJ, precuneus, and MPFC (16, 35). Most of our life is spent interacting with personally familiar individuals, and quick recognition and activation of person knowledge are prerequisites for appropriate and effective social interactions. If we are to understand how the face processing system works outside of artificial laboratory settings, we need to study the human brain with the stimuli it evolved to process for survival: socially relevant, personally familiar others.

## Materials and Methods

### Participants.

Fourteen participants (6 female, mean age 27.42 y ± 1.74 SD) took part in the experiment. All had normal or corrected-to-normal vision. Participants were graduate students in the Psychology and Brain Sciences Department at Dartmouth College. The study was approved by the Dartmouth Committee for the Protection of Human Subjects. All participants provided written informed consent to the study. The participants in this experiment also took part in a movie-watching experiment (*The Grand Budapest Hotel*). The movie-watching dataset is publicly available, and we refer the reader to the publication for more details (20).

### Stimuli.

Stimuli for the familiar face perception experiment were faces of eight different individuals in five different head views each (Fig. 1*A*). Four graduate students (two female) in the same department as the participants served as models for the personally familiar faces. Four undergraduates (two female) served as models for the unfamiliar faces (see *Procedure*). (The undergraduate models graduated before the study was conducted; thus, participants were not familiar with them.) All models were Caucasian. Short videos and still pictures of each person were taken in the laboratory. The short videos were taken while the experimenter explained the procedure to the models and asked them to look around the room, which ensured a natural transition to different head views. Each video was shown to the participants of the fMRI experiment without the audio. The videos covered the person’s head and shoulders, and all models wore a black T-shirt. During the recordings, models were conversing naturally and listening to the experimenter. These recordings (without the audio) were used during a behavioral training session to visually familiarize participants with the unfamiliar individuals. All models provided written informed consent to allow the use of their images for research and in publications.

Still face images were taken with five different head views: left and right full profile, left and right half profile, and full-frontal view (Fig. 1*B*). To ensure consistent image quality, all pictures were taken in the same studio with identical equipment and lighting conditions. All still images were cropped and included the hair. Each image was scaled to a resolution of 500 × 500 pixels, gray scaled, and matched in average luminance and contrast using the SHINE Toolbox (36).

fMRI data were also collected while participants viewed the final 50 min of *The Grand Budapest Hotel*. Participants watched the first part of the movie right before the scanning session. These data are part of a larger, publicly available movie-watching dataset, and we refer the reader to the publication for more details (20).

### Procedure.

Each participant took part in one behavioral session and three fMRI sessions. During the behavioral session, participants completed a training task to become visually familiar with four unfamiliar identities. During two fMRI sessions, participants performed a one-back repetition detection task on identity for visually familiar and personally familiar faces (Fig. 1*A*). A questionnaire was sent to the participants after the experiment to quantify how familiar they were with the personally familiar individuals shown in the experiment. In the third fMRI session, participants watched the final 50 min of *The Grand Budapest Hotel* (20).

Before the behavioral training session, we verified that participants did not know any of the identities used for the visually familiar condition. In the behavioral training session, participants viewed four 15 s videos for each identity (no audio). Then, participants performed a face identity-matching task. Each trial consisted of two stimuli separated by a 0.5 s interstimulus interval. Stimuli were still images of the four identities with different head views (presented for 1 s) or 1 s video clips randomly selected from the 15 s video clips. Participants reported if the identity was the same or different using a keyboard. There were 360 trials in total, with matching identities in half of the trials. Participants were shown their accuracy as feedback every 30 trials (12).

Participants underwent two separate fMRI sessions on separate days (max 2 d between sessions). While they underwent anatomical scans, participants were shown all 40 images that would be used in the experiment: 4 identities × 5 head views × 2 familiarity types (visually familiar, personally familiar). After the anatomical scan and a gradient-echo fieldmap scan, participants underwent 10 functional runs for the main task and one functional run for a localizer. In each functional run, faces from only one type of familiarity were shown, and the order of the runs was counterbalanced across sessions (the first session started with a “personally familiar face” run, and the second session started with a “visually familiar face” run, alternating conditions). Thus, the familiarity of the stimuli was blocked in each run. The sixth run was always a localizer run. (Data from these localizer runs were not used for the analyses reported here.)

Each run had 63 trials (60 stimulus trials and 3 fixation trials). Each stimulus trial was 5 s long and started with a stimulus image presented for 500 ms followed by a 50-ms black screen, repeated three times, and followed by 3,400 ms fixation (Fig. 1*A*). In each trial, the image size was jittered (±50 pixels equivalent to 1.25° variations). The three repetitions on each trial were of the same identity and head view but with the location jittered (±10 pixel variations in the horizontal and vertical location). Each face image subtended ∼12.5° of visual angle. Participants performed a one-back repetition detection task based on identity, pressing a button with the right index finger for “same” and the right middle finger for “different” (12). All runs started and ended with 15 s of a white fixation cross on a black background.

To minimize carry-over effects, the trial sequence was created by generating Type-1, Index-1 (T1I1) sequences (37, 38) (https://cfn.upenn.edu/aguirre/wiki/public:t1i1_sequences) as follows. First, 10,000 T1I1 sequences with 21 labels (20 trial conditions plus a null trial) were generated and their efficiency (39) was computed (using the tool from https://cfn.upenn.edu/aguirre/wiki/doku.php?id=public:selection_of_efficient_type_1_index_1_sequences). The efficiency calculation was based on matrices that indicated the relationship between pairs of stimuli. We used three such matrices: identity (1 for same identity and 0 otherwise), head-view (1 for same head view and 0 otherwise), and mirror-symmetry (for example, 1 for left and right profile views). Then, we selected the top two sequences, one for personally familiar faces and one for visually familiar faces. Because each T1I1 sequence with 21 labels is 21^2^ + 1 = 442 trials long, we generated a longer sequence by appending another T1I1 sequence that started with the same label. The final sequence was then broken into 10 subsequences with 63 trials for each run.

An important property of T1I1 sequences is that every 21 trials all labels are presented. Thus, in each run, each image was presented exactly three times, with three blank trials. An extra trial was added at the beginning of each run, either repeating the first image (for the first run) or repeating the last trial of the previous run (for the remaining nine runs). The sequence order was counterbalanced across participants by inverting the original sequences every other participant (an inverted T1I1 sequence is a T1I1 sequence).

### Imaging Parameters.

All functional and structural images were acquired using a 3T Siemens Magnetom Prisma MRI scanner (Siemens) with a 32-channel phased-array head coil at the Dartmouth Brain Imaging Center. Functional blood-oxygenation level-dependent images were acquired in an interleaved fashion using gradient-echo echo-planar imaging with prescan normalization, fat suppression, a multiband (i.e., simultaneous multislice) acceleration factor of 4 (using blipped CAIPIRINHA), and no in-plane acceleration (i.e., in-plane acceleration factor of 1): repetition time/echo time TR/TE = 1,250/33 ms, flip angle = 64°, resolution = 2.5 mm^3^ isotropic voxels, matrix size = 96 × 96, field of view (FoV) = 240 × 240 mm, 56 axial slices with full brain coverage and no gap, and anterior–posterior phase encoding. At the beginning of each run, three dummy scans were acquired to allow for signal stabilization. A gradient-echo fieldmap scan was acquired at the beginning of each scanning session for EPI distortion correction.

A T1-weighted structural scan was acquired using a high-resolution single-shot Magnetization Prepared - RApid Gradient Echo (MP-RAGE) sequence with an in-plane acceleration factor of 2: repetition time/echo time/inversion time TR/TE/TI = 2,300/2.32/933 ms, flip angle = 8°, resolution = 0.9375 × 0.9375 × 0.9 mm voxels, matrix size = 256 × 256, FoV = 240 × 240 × 172.8 mm, 192 sagittal slices, ascending acquisition, anterior–posterior phase encoding, no fat suppression, and 5 min 21 s total acquisition time. A T2-weighted structural scan was acquired with an in-plane acceleration factor of 2 using GRAPPA: TR/TE = 3,200/563 ms, flip angle = 120°, resolution = 0.9375 × 0.9375 × 0.9 mm voxels, matrix size = 256 × 256, FoV = 240 × 240 × 172.8 mm, 192 sagittal slices, ascending acquisition, anterior–posterior phase encoding, no fat suppression, and 3 min 21 s total acquisition time.

### Data Preprocessing.

Anatomical and functional data were preprocessed using fMRIPrep version 1.0.3 (40). Functional data were slice-time corrected, motion corrected, distortion corrected, and projected to the standard surface template *fsaverage6*, consisting of 40,962 nodes for each hemisphere. Physiological noise components were estimated using CompCor (41). (See the *SI Appendix*, *Supplementary Methods* for more information about the preprocessing steps.) No additional smoothing was performed in any of the analyses.

### Hyperalignment.

We estimated hyperalignment parameters with whole-brain searchlight hyperalignment (18, 19). Hyperalignment was based on movie-watching data that was collected previously. The dataset is publicly available, and we refer the reader to the associated publication for more details (20). Transformation matrices were determined for discal searchlights of radius 20 mm, ignoring nodes in the medial wall.

Functional data for each participant was then projected into the reference participant’s space by combining the individual projection matrix to the common model space with the transpose of the reference participant’s projection matrix. This transformation was performed as a single step and was applied to the z-scored preprocessed functional runs. The hyperaligned functional runs were then used for all subsequent analyses.

### GLM Modeling.

 Before fitting a general linear model (GLM), we regressed out the motion parameter estimates and the first six physiological noise components estimated by CompCor. In addition, the signal was high-pass filtered at a frequency of 0.0066 Hz. This preprocessing step was performed as a single operation using *3dTProject* in AFNI.

We modeled the task data for multivariate analyses using a canonical hemodynamic response function (HRF) (BLOCK response in AFNI) convolved for the duration of the stimulus (1.6 s). Each image was modeled separately within each run, yielding 400 regressors of interest (10 runs × 2 types of familiarity × 4 identities × 5 head-views). We also modeled participants’ responses (whenever participants responded “same”), as well as the first repeated trial in each run as a single condition. Additional nuisance regressors included polynomial changes up to third order. A single model was fitted with all conditions using *3dDeconvolve* in AFNI. The resulting t-values associated with the regressors of interest were used for MVPC (42).

### Definition of ROIs in the Core and Extended System.

Face-responsive ROIs were determined after hyperalignment in the reference participant’s space with data collected for another experiment. In separate scanning sections, 21 participants (including the 14 reported here) performed four runs of a dynamic functional localizer (see refs. 43 and 44 and https://github.com/mvdoc/pitcher_localizer for more details).

The first localizer run for each participant was hyperaligned and mapped back to the reference participant’s space. We then modeled the corresponding 21 hyperaligned localizer runs using *3dREMLFit* in AFNI. Each condition (faces, objects, scrambled objects, scenes, and bodies) was modeled using a standard HRF response convolved with a boxcar function. Nuisance regressors included polynomial changes up to third order, as well as button presses.

To create ROIs for face-responsive areas, we followed a two-step process. In the first step, we visualized the contrast “Face vs. Other categories” in SUMA (45). The contrast map was thresholded at t = 1.96 (corresponding to *P* < 0.05, two-sided). If no nodes surpassed the threshold for one of the ROIs, the threshold was lowered to t = 1.65 (corresponding to *P* < 0.05, one-sided). Then, we manually selected center nodes near or at peaks of activation for the following nine face-responsive ROIs: OFA, pFFA, mFFA, aFFA, ATL, pSTS, mSTS, anterior STS, and IFG.

In the second step, the center nodes were automatically refined by selecting the node with the maximum t-value in a discal neighborhood of radius 3 mm centered around the peaks determined in the first step. The final face-responsive ROIs were created by selecting all nodes within a 15 mm radius from the center nodes. Nodes that belonged to more than one ROI were assigned to the closest ROI according to the geodesic distance on the surface. Thus, all ROIs were nonoverlapping.

Extended system ROIs were generated by merging anatomical parcels from the multimodal parcellation developed by the Human Connectome Project (46). The corresponding parcels were individuated manually. We created four ROIs for areas of the extended system: MPFC, TPJ, precuneus, and insula.

### MVPC.

All MVPC analyses were performed on hyperaligned data projected to the template surface *fsaverage6*. All analyses were implemented in Python and PyMVPA (47). MVPC used a linear SVM classifier (48). The SVM classifier used the default soft-margin option in PyMVPA, which automatically scales the regularization parameter according to the norm of the data.

Between-subject identity classification was performed by nested cross-validation across participants and head views (Fig. 1*B*). First, the hyperaligned data from each participant were averaged across runs, yielding 20 samples (4 identities × 5 head views) for each participant and type of familiarity. Second, the data were split into a training set that consisted of data from n − 1 participants, and a test set with data from the left-out nth participant. This step corresponds to cross-validation across participants. Third, a classifier was trained on samples from the training set associated with the four identities in four head views. The classifier was tested on samples from the test set associated with the four identities in the left-out head view. This step corresponds to cross-validation across head views. This process was repeated exhaustively for all head views and participants and resulted in 5 × 14 = 70 folds. As a result of this process, the classifier was trained and tested on completely independent data, both at the level of participants and stimuli (Fig. 1*B*).

MVPC analyses were performed both whole-brain and within ROIs. For whole-brain analyses, we centered surface searchlights on each node and included all nodes within a cortical disk of radius 10 mm. Classification accuracies from each searchlight were placed into their center surface nodes, resulting in one accuracy map for each cross-validation fold. For ROI analyses, nodes associated with each ROI were used instead of searchlights.

Statistical thresholding was performed with permutation testing. First, all MVPC analyses were repeated 100 times by randomly shuffling identity labels within each participant and head view independently. This shuffling maintained the same proportion of labels in each participant and head view as the original dataset. Then, to create an empirical null distribution, we bootstrapped (random sampling) 70 cross-validation folds (5 head views × 14 participants) from the permuted analyses and averaged across these folds. This process was repeated 10,000 times. For each searchlight or ROI, we then estimated the empirical *P* value by counting how many times the null distribution exceeded the accuracy value of the original unpermuted dataset (adding 1 to both numerator and denominator to adjust for bias; see ref. 49). This corresponds to a one-sided permutation test. The Benjamini-Hochberg (50) False Discovery Rate procedure (FDR correction) was used to correct the resulting *P* values for multiple comparisons.

To compute the difference in classification accuracy between personally familiar identities and visually familiar identities, we first averaged the accuracy values from the 70 cross-validation folds for the two conditions (personal and visual) independently. Then, in each of the two resulting maps, we set accuracy values below chance level to 25% (*SI Appendix*, Fig. S3). Values below chance level are caused by noise in the data, and thus they are not meaningful, but they can inflate the estimate of the difference. Finally, the difference map between the two conditions (personal–visual) was computed. Statistical testing was performed with permutation testing. Permuted cross-validation folds were bootstrapped (random sampling) for the two conditions independently, an average map across cross-validation folds was computed for each condition, accuracy values below chance level were set to 25%, and a permuted difference map was computed. This process was repeated 10,000 times to generate a null distribution of difference values for each searchlight or ROI. An empirical two-sided *P* value was computed by counting how many times the absolute value of the null distribution exceeded the absolute value of the original unpermuted difference map (adding 1 to both numerator and denominator to adjust for bias; see ref. 49). The resulting *P* values were then FDR-corrected using the Benjamini-Hochberg procedure (50).

## Data Availability

Data from the fMRI face perception experiment is publicly available on OpenNeuro at https://openneuro.org/datasets/ds003834 (52). The movie-watching data of *The Grand Budapest Hotel* is publicly available at https://openneuro.org/datasets/ds003017 (51). Code associated with this work is available on GitHub at https://github.com/mvdoc/identity-decoding.
